# Effect of Stem Cell Therapy on Bone Mineral Density: A Meta-Analysis of Preclinical Studies in Animal Models of Osteoporosis

**DOI:** 10.1371/journal.pone.0149400

**Published:** 2016-02-16

**Authors:** Feng Li, Changlin Zhou, Liang Xu, Shuqing Tao, Jingyi Zhao, Qun Gu

**Affiliations:** 1 Department of Bone joint surgery, the Second Affiliated Hospital of Harbin Medical University, Harbin, Heilongjiang Province, 150086, China; 2 Department of emergency surgery, the Second Affiliated Hospital of Harbin Medical University, Harbin, Heilongjiang Province, 150086, China; 3 Heilongjiang academy of traditional Chinese medicine, Harbin, Heilongjiang Province, 150086, China; Nanjing Medical University, CHINA

## Abstract

**Background:**

Preclinical studies of the therapeutic role of stem cell based therapy in animal models of osteoporosis have largely yielded inconsistent results. We performed a meta-analysis to provide an overview of the currently available evidence.

**Methods:**

Pubmed, Embase and Cochrane Library databases were systematically searched for relevant controlled studies. A random-effect model was used for pooled analysis of the effect of stem cell based therapy on bone mineral density (BMD). Stratified analyses were performed to explore the effect of study characteristics on the outcomes.

**Results:**

Pooled results from 12 preclinical studies (110 animals in stem cell treatment groups, and 106 animals in control groups) indicated that stem cell based treatment was associated with significantly improved BMD (standardized mean difference [SMD] = 1.29, 95% Confidence Interval [CI]: 0.84–1.74, *P* < 0.001) with moderate heterogeneity (Cochrane’s Q test: *P* = 0.02, I^2^ = 45%) among the constituent studies. Implantation of bone marrow cells, bone marrow mesenchymal stem cells, adipose-derived stem cells, and human umbilical cord blood-derived CD34+ cells, were all associated with improved BMD as compared to that in the controls (*P* < 0.05 for all); the only exception being the use of embryonic stem cell transplantation (*P* > 0.05). Egger’s test detected potential publication bias (*P* = 0.055); however, ‘trim and fill’ analysis yielded similar results after statistically incorporating the hypothetical studies in the analysis (SMD = 1.24, 95% CI: 0.32–2.16, *P* < 0.001).

**Conclusions:**

Stem cell transplantation may improve BMD in animal models of osteoporosis. Our meta-analysis indicates a potential therapeutic role of stem cell based therapy for osteoporosis, and serves to augment the rationale for clinical studies.

## Introduction

Osteoporosis has been recognized as a systemic disease which is characterized by the continuous loss of bone mass and subsequent degeneration and deterioration of bone microarchitecture [1]. Patients with osteoporosis are at a higher risk of bone fractures, and particularly among the elderly who are vulnerable to falling [2]. The prevalence of osteoporosis is relatively high in both the developed and the developing countries. An estimated 200 million people worldwide are suffering from osteoporosis [3]. In China, about 1 in 5 persons aged over 50 years are believed to have osteoporosis [4]. Currently, the preventive and therapeutic strategies for patients with osteoporosis are based on the supplementation of calcium and vitamin D, use of pharmacological agents which inhibit bone resorption such as bisphosphonates, and occasionally the administration of calcitonin [5]. Although bisphosphonates have been shown to reduce the risk of osteoporosis [6], clinical use of these medications has been limited by its potential to cause serious side effects, such as osteonecrosis of the jaw and atypical femoral fractures [7]. Therefore, development of novel treatment strategies for osteoporosis is of great clinical significance.

The key pathophysiological mechanism in osteoporosis is the imbalance between the bone formation and the bone resorption. In addition to targeting bone resorption, stimulation and restoration of bone formation is also considered to be an effective therapeutic strategy for osteoporosis [8]. Bone formation is mainly mediated by the osteoid-secreting osteoblasts. However, this physiological repair process is tempered in the elderly due to the limited regenerative ability and the decreased numbers of the osteoblast progenitor cells, termed bone marrow mesenchymal stem cells (BMSCs) [9, 10]. Stem cell transplantation has been suggested as a potential therapeutic strategy for patients with osteoporosis [11]. Indeed, several experimental studies have been performed in animal models of osteoporosis to evaluate the therapeutic effect of stem cell plantation [12–23]; the results of these preclinical studies, however, have not always been consistent. Moreover, to the best of our knowledge, no clinical trials in human of stem cell based treatment for osteoporosis have been reported and the literature focusing on the potential role of stem cell transplantation in animal models of osteoporosis is rare [24].

We performed a meta-analysis of animal studies that assessed the effect of stem cell transplantation on bone mineral density (BMD), a reliable parameter of bone mass that reflects the severity of osteoporosis [25]. Moreover, we also tried to explore whether the stem cells from different sources, and administered using different delivery routes, work differently in this respect.

## Methods

The primary aim of the meta-analysis was to evaluate the influence of stem cell based therapy on BMD in animal models of osteoporosis. We followed the Preferred Reporting Items for Systematic reviews and Meta-Analyses (PRISM) [26], and the Cochrane Handbook guidelines [27] for the conduct and reporting of the study.

### Literature search

We performed a systematic search for relevant studies in Pubmed, Embase, and the Cochrane Library (Cochrane Center Register of Controlled Trials) databases. The key words used for literature search were: "stem cells", "progenitor cells", "bone marrow cells", paired with "bone mineral density", "BMD" AND "osteoporosis", "osteopenic". The final search of the database was performed on August 10th, 2015. The references cited in the retrieved articles were manually assessed to widen the yield of studies.

### Study subjects

Studies were included if they met all of the following criteria: 1) published as a full-length article in peer-reviewed journals in any language; 2) reported as a randomized or a cohort study with a control group; 3) animal models of osteoporosis were assigned to either a group for the topical or systemic transplantation of stem cells (either bone marrow derived or peripheral blood derived) or a control group (no treatment or placebo); 4) reported on the change of BMD in both study groups, or the relevant data was available for calculation. Trials that investigated only transfected or genetically engineered stem cells that altered cell behavior were excluded; however, studies using reporter genes (solely for stem cell imaging purposes) were included. Abstracts, reviews, case reports, and other studies not designed as randomized or cohort studies with control groups were excluded from the purview of this meta-analysis.

### Data extraction and quality assessment

Two authors independently performed the literature search, data extraction, and quality assessment according to the inclusion criteria. Discrepancies if any were resolved by consensus. Data pertaining to study design (randomized or cohort), details of the animal model, stem cell treatment modalities (source of stem cells, quantity, delivery method used for transplantation, and the follow-up duration), and the BMD measurement methods were extracted for further analyses. Studies in which stem cells were transplanted using more than one dose regimen or those that included multiple intervention groups with varying durations of follow-up, multiple comparisons were included in the meta-analysis independently. In case of studies with incomplete data the corresponding authors were contacted for access to the unpublished data.

Quality assessment of the included studies was performed using modified Jadad score criteria since the standard guidelines for quality assessment of clinical trials are not universally applicable to preclinical studies [28]. The modified Jadad scale criteria which were used to assess selection, performance and detection bias included the followings: (1) randomization; (2) description of randomization; (3) adequate allocation; (4) blinding of the operator and (5) blinding of the outcome analysis. Trials scoring 1 point were deemed to be of low quality, and 4–5 points were considered indicative of high quality.

### Statistical analysis

The primary outcome for this meta-analysis was the change in BMD, from baseline to the study endpoint, in response to stem cell based therapy. The pooled effect is presented as standard mean difference (SMD) with 95% confidence intervals (CI). Inter-study heterogeneity was formally evaluated using Cochrane's test; an associated *P* value of < 0.10 was considered as indicating significant heterogeneity. The I^2^ statistic, which describes the percentage of total variation across studies that is attributable to heterogeneity rather than to chance, was also calculated; a value of I^2^ > 50% was considered indicative of a significant heterogeneity [29]. If multiple experimental groups were compared with a single control group within one study, the number of animals in the control group was divided equally by the number of experimental groups. A random-effect model was used to estimate the overall effect instead of a fixed-effect model, given that the former is a more conservative approach that takes into account that study heterogeneity can vary beyond chance, thus providing more generalizable results [27]. Predefined stratified analyses were performed to explore the possible influence of the study characteristics (study design, animal model, source and amounts of stem cells and the delivery method used, the BMD measurement methods and duration of follow-up) on the pooled outcome. Median values of continuous variables were used as cutoff values for grouping studies. The stability of the results was assessed by sensitivity analysis after excluding certain studies. Furthermore, potential publication bias was assessed with funnel plot and Egger regression asymmetry test [30]. Moreover, we performed the nonparametric ‘‘trim and fill” procedure [27] to further assess the possible effect of publication bias on the results of our meta-analysis. The ‘‘trim and fill” method considers the possibility of hypothetical “missing” studies that might exist, inputs their potential effects, and recalculates a synthesized effect that incorporates the hypothetical missing studies as though they actually existed. *P* values were two-tailed and statistical significance was set at 0.05. RevMan (Version 5.1; Cochrane, Oxford, UK) and Stata (Version 12.0; Stata, College Station, TX) software were used for the meta-analysis.

## Results

### Search results

**Fig 1:**
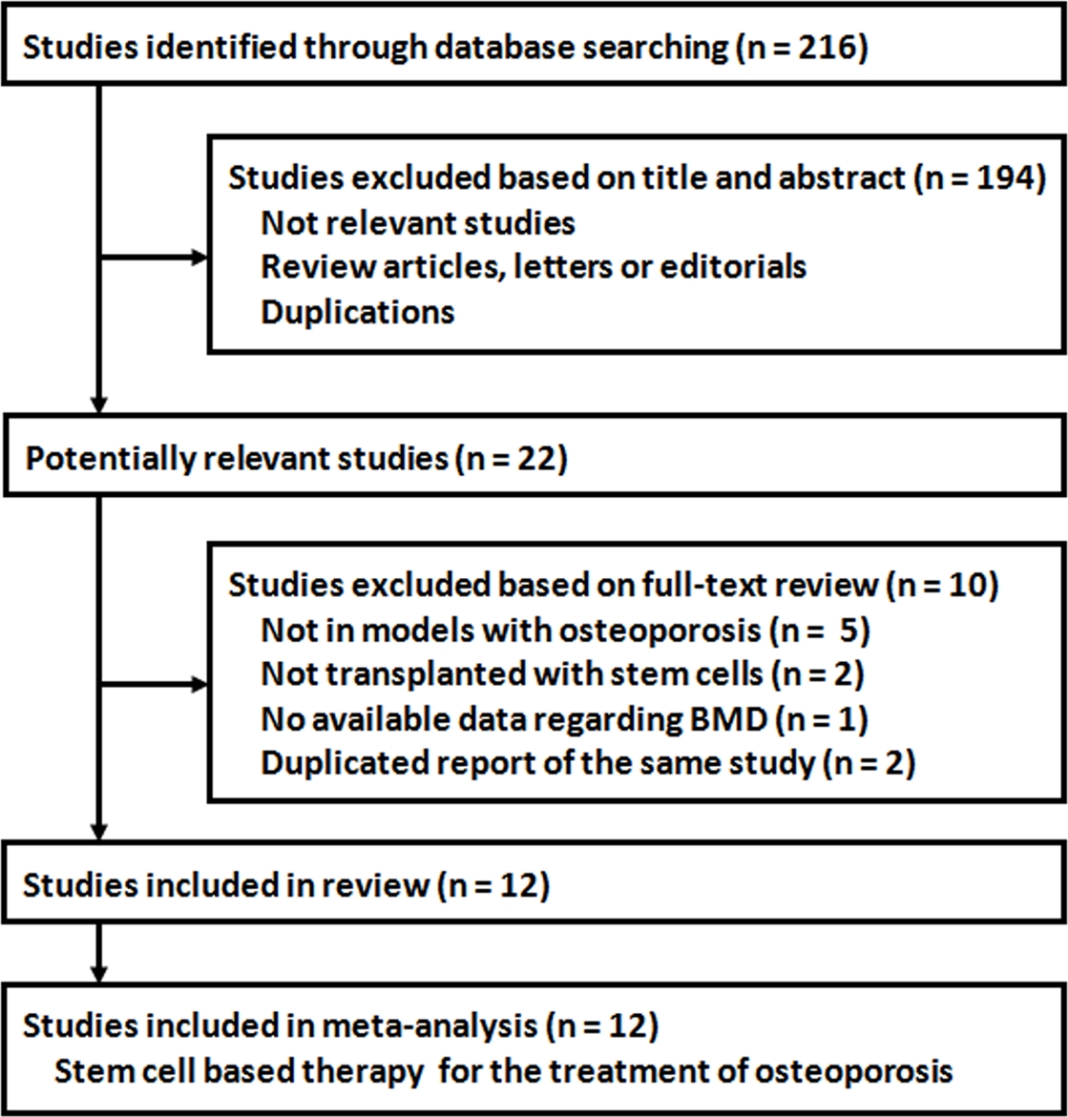
Flow diagram illustrating the study selection criteria for the meta-analysis. *BMD*, *Bone mineral density*

A flowchart illustrating the literature search and study selection is presented as **Fig 1**. A total of 216 articles were retrieved after initial search of the databases. A total of 194 articles were excluded after screening the titles and abstracts, mainly because these studies were not considered to be of relevance to the current meta-analysis, or they were literature reviews, and duplicate reports. For the 22 potentially relevant studies, 10 were further excluded after the review of the full-texts because 5 studies did not involve animal models of osteoporosis, two studies did not involve stem cell therapy, two papers represented duplication of the same studies, and one study did not report on BMD outcomes. Finally, 12 studies [12–23] investigating the therapeutic role of stem cell based intervention on BMD in osteoporotic animals were included in the meta-analysis.

### Study characteristics

The key characteristics of the 12 included studies [12–23] are presented in **Table 1**. Of note, the study by Uejima et al [13], Yu et al [19], and Jeong et al [21] included multiple comparisons with different amounts of stem cells and had variable follow-up durations. These comparisons were included in the meta-analysis independently. Overall, this meta-analysis included 19 comparisons (110 animals in the stem cell treatment group, and 106 animals in the control group) of the effect of stem cell based therapy on BMD.

**Table 1:** Baseline characteristics of the studies included in the meta-analysis.

Author year	Study design	Animal	Experimental model used	Stem cell origin	Stem cell type	Cell amount	Delivery method	Measures for rejection	Times of injection	BMD location	BMD measurement	Follow-up duration (weeks)
Ichioka 2002 [12]	Cohort	Mice	Senescence accelerated mice	Allogeneic	BMCs	3×10^7^	IBM	Irradiation	1	femur	Soft X-ray	32
Uejima 2008a [13]	RCT	Rats	Ovariectomy	Syngeneic	BMSCs	1×10^5^	IBM	NR	1	femur	Dualenergy X-ray	8
Uejima 2008b [13]	RCT	Rats	Ovariectomy	Syngeneic	BMSCs	1×10^5^	IBM	NR	1	femur	Dualenergy X-ray	24
Uejima 2008c [13]	RCT	Rats	Ovariectomy	Syngeneic	BMSCs	2×10^5^	IBM	NR	2	femur	Dualenergy X-ray	24
Cho 2009 [14]	RCT	Mice	Ovariectomy	Syngeneic	BMSCs	1.5×10^6^	ITV	NR	2	femur	Dualenergy X-ray	8
Feng 2010 [15]	RCT	Mice	Ovariectomy	Allogeneic	BMCs	1x10^7^	IBM	Irradiation	1	proximal tibia	Peripheral qCT	12
Tao 2011 [16]	RCT	Rats	Glucocorticoid-induced osteoporosis	Syngeneic	ADSCs	3×10^6^	ITV	NR	1	femur	Dualenergy X-ray	4
Liu 2012a [18]	Cohort	Mice	Ovariectomy	Syngeneic	ADSCs young	1x10^6^	IBM	NR	1	femur	Dualenergy X-ray	16
Liu 2012b [18]	Cohort	Mice	Ovariectomy	Syngeneic	ADSCs aged	1×10^6^	IBM	NR	1	femur	Dualenergy X-ray	16
Yu 2012a [19]	RCT	Rats	Ovariectomy	Syngeneic	BMSCs	1.2×10^7^	IBM	NR	1	femur	Dualenergy X-ray	4
Yu 2012b [19]	RCT	Rats	Ovariectomy	Syngeneic	BMSCs	1.2×10^7^	IBM	NR	1	femur	Dualenergy X-ray	12
Aggarwal 2012 [17]	Cohort	Mice	Glucocorticoid-induced osteoporosis	Xenogenic	HUCBDCs	5×10^5^	ICV	NR	1	femur	High-resolution Micro CT	4
Yu 2013 [20]	Cohort	Mice	Ovariectomy	Syngeneic	BMSCs	1×10^6^	ITV	NR	1	femur	High-resolution Micro CT	4
Taiani 2014 [22]	Cohort	Mice	Ovariectomy	Syngeneic	ESCs	2×10^5^	IBM	NR	1	tibia	High-resolution Micro CT	4
Ye 2014[23]	RCT	Rabbits	Ovariectomy	Syngeneic	ADSCs	5×10^6^	IBM	NR	1	femur	High-resolution Micro CT	12
Jeong 2014a [21]	RCT	Rats	Ovariectomy	Syngeneic	ADSCs	4×10^6^	ITV	NR	2	femur	High-resolution Micro CT	5
Jeong 2014b [21]	RCT	Rats	Ovariectomy	Syngeneic	ADSCs	4×10^6^	ITV	NR	2	femur	High-resolution Micro CT	6
Jeong 2014c [21]	RCT	Rats	Ovariectomy	Syngeneic	ADSCs	4 × 10^6^	ITV	NR	2	femur	High-resolution Micro CT	7
Jeong 2014d [21]	RCT	Rats	Ovariectomy	Syngeneic	ADSCs	4×10^6^	ITV	NR	2	femur	High-resolution Micro CT	8

The study by Uejima 2008 included 3 comparisons with different amounts of stem cells and follow-up durations; the study by Liu 2012 included 2 comparisons with different sources of ADSCs; Yu 2012 included 2 comparisons with different follow-up durations; and the study by Jeong 2014 included 4 comparisons with different follow-up durations. These comparisons were included in the meta-analysis independently. *RCT*, *randomized controlled trial; BMCs*, *bone marrow cells; BMSCs*, *bone marrow mesenchymal stem cells; ADSCs*, *adipose-derived stem cells; HUCBDCs*, *human umbilical cord blood-derived CD34+ cells; ESCs*, *embryonic stem cells; IBM*, *intra-bone marrow; ITV*, *intra-tail venous; ICV*, *intra-cardio ventricular; CT*, *computed tomography; qCT*, *quantitative CT; BMD*, *bone mineral density; NR*, *not reported*.

These studies were all preclinical studies in small animal models of osteoporosis (mice [12, 14, 15, 17, 18, 20, 22], rats [13, 16, 19, 21] and rabbits [23]). The modeling of osteoporosis was induced by ovariectomy in most of the studies [13–15, 18–23]. The experimental protocols for spontaneous osteoporosis in senescence accelerated mice [12] and glucocorticoid induced osteoporosis [16, 17] were also used. The stem cells used in the treatment group included bone marrow cells (BMCs) [12, 15], bone marrow mesenchymal stem cells (BMSCs) [13, 14, 19, 20], adipose-derived stem cells (ADSCs) [16, 18, 21, 23], human umbilical cord blood-derived CD34+ cells (HUCBDCs) [17] and embryonic stem cells (ESCs) [22], with total amounts of the transplanted cells of 1x10^5^ to 3x10^7^. For the included 19 comparisons, 16, 2, and 1 studies used syngeneic [13, 14, 16, 18–23], allogeneic [12, 15], and xenogenic [17] transplantation, respectively. Pretreatment irradiations were performed in 2 of the comparisons [12, 15], while others did not report any strategies for ameliorating the potential rejection. Stem cell transplantation was achieved mainly by intra-bone marrow (IBM) injection [12, 13, 15, 18, 19, 22, 23] and intra-tail venous (ITV) injection [14, 16, 20, 21], while intra-cardio ventricular (ICV) injection was used in one study [17]. Both the x-ray based and the computed tomography (CT)-based measurements were factored in the evaluation of BMD. The follow-up duration in the included studies varied from 4 to 32 weeks.

### Quality assessment

Details of the quality assessment of the included studies are summarized in **Table 2**. Of the included studies, 7 were randomized controlled studies [13–16, 19, 21, 23], while the other 5 were cohort studies with controls [12, 17, 18, 20, 22]. The methodological quality of the included trials was poor in general, with 5 studies awarded 3 points [13, 15, 20, 21, 23], 2 studies awarded 2 points each [14, 16], and 5 awarded a score of 1 point [12, 17, 18, 20, 22], as evaluated by modified Jadad Score. The poor quality of the studies, in large part, reflects the preclinical nature of the study.

**Table 2:** Quality assessment of studies included in the meta-analysis using the modified Jadad Score.

Study	Random	Randomization methods	Adequate allocation	Blinding of the operator	Blinding of the outcome analysis	Total score
Ichioka 2002 [12]	0	0	1	0	0	1
Uejima 2008 [13]	1	1	1	0	0	3
Cho 2009 [14]	1	0	1	0	0	2
Feng 2010 [15]	1	1	1	0	0	3
Tao 2011 [16]	1	0	1	0	0	2
Liu 2012 [18]	0	0	1	0	0	1
Yu 2012 [19]	1	1	1	0	0	3
Aggarwal 2012 [17]	0	0	1	0	0	1
Yu 2013 [20]	0	0	1	0	0	1
Taiani 2014 [22]	0	0	1	0	0	1
Ye 2014 [23]	1	1	1	0	0	3
Jeong 2014 [21]	1	1	1	0	0	3

### Meta-analysis

**Fig 2:**
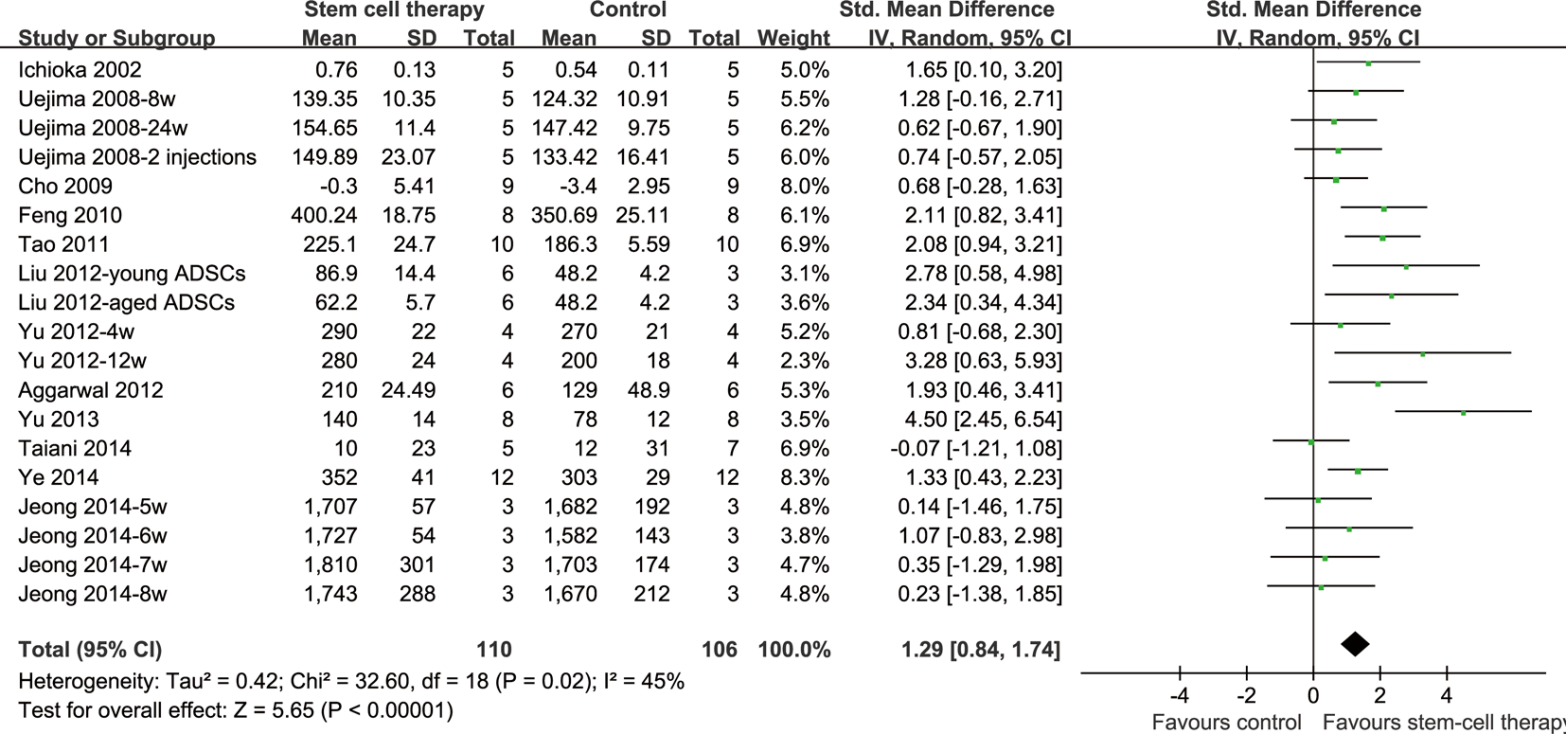
Forest plot of the meta-analysis of standardized mean difference of bone mineral density by study group (stem cell based treatment- vs. control group). *BMD, Bone mineral density*; *SD, standard deviation; CI, confidence interval; df, degrees of freedom*

Pooled analysis of data comprising of 19 inter-group comparisons generated from 12 original studies was performed. Meta-analysis using a random effect model indicated that stem cell based treatment was associated with significantly improved BMD (SMD = 1.29, 95% CI: 0.84–1.74, *P* < 0.001, **Fig 2**). Moderate heterogeneity among the included studies was detected (Cochrane’s Q test: *P* = 0.02, I^2^ = 45%, **Fig 2**).

### Stratified analyses

To further explore the potential influence of study methods on the beneficial effect of stem cell based therapy on BMD, we further performed stratified analyses according to some of the study features (**Table 3**). The beneficial effects of stem cell based therapy on BMD were remarkable in both randomized studies (SMD = 1.09, 95% CI: 0.70–1.48, p < 0.001) and the cohort studies (SMD = 2.04, 95% CI: 0.76–3.32, p = 0.002). Transplantation of BMCs (SMD = 1.92, 95% CI: 0.93–2.92), BMSCs (SMD = 1.39, 95% CI: 0.52–2.27), ADSCs (SMD = 1.26, 95% CI: 0.63–1.88) and HUCBDCs (SMD = 1.93, 95% CI: 0.46–3.41) were all associated with significantly improved BMD as compared to that observed in controls (**Table 3**). However, transplantation with ESCs did not significantly affect the BMD in animals of osteoporosis (SMD = -0.07, 95% CI: -1.21–1.08; *p* = 0.91). Other study characteristics, such as the animals used and the modeling methods, the amounts and route of stem cell delivered, the origins of the stem cells, the measurements of BMD, and the follow-up duration, appeared to have no significant influence on the benefits of stem cell transplantation on BMD (**Table 3**).

**Table 3:** Stratified analyses for the effects of stem-cell therapy on bone mineral density in animal models of osteoporosis.

	Number of group comparisons	SMD [95% CI]	*P* for subgroup outcomes	I^2^	*P* for subgroup interactions
**Study design**					
RCTs	13	1.09 [0.70, 1.48]	< 0.001	9%	0.16
Cohorts	6	2.04 [0.76, 3.32]	0.002	72%	
**Animals**					
Mice	8	1.79 [0.88, 2.71]	< 0.001	67%	0.30
Rats	10	0.99 [0.50, 1.48]	< 0.001	6%	
Rabbits	1	1.33 [0.43, 2.23]	0.004	—	
**Models**					
Accelerated senescence	1	1.65 [0.10, 3.20]	0.04	—	0.26
Ovariectomy	16	1.17 [0.66, 1.68]	< 0.001	47%	
Glucocorticoid	2	2.02 [1.12, 2.92]	< 0.001	0%	
**Stem cells**					
BMCs	2	1.92 [0.93, 2.92]	< 0.001	0%	0.10
BMSCs	7	1.39 [0.52, 2.27]	0.002	60%	
ADSCs	8	1.26 [0.63, 1.88]	< 0.001	27%	
HUCBDCs	1	1.93 [0.46, 3.41]	0.01	—	
ESCs	1	-0.07 [-1.21, 1.08]	0.91	—	
**Cell amount**					
> 1×10^6^	11	1.20 [0.73, 1.66]	< 0.001	20%	0.49
≤ 1×10^6^	8	1.56 [0.64, 2.48]	< 0.001	65%	
**Delivery methods**					
IBM	11	1.27 [0.75, 1.78]	0.01	29%	0.69
ITV	7	1.21 [0.25, 2.16]	0.01	66%	
ICV	1	1.93 [0.46, 3.41]	< 0.001	—	
**BMD measurements**					
x -ray based	11	1.41 [0.94, 1.89]	< 0.001	18%	0.51
CT—based	8	1.08 [0.23, 1.94]	0.01	64%	
**Follow-up duration**					
> 8 wks	8	1.52 [0.98, 2.06]	< 0.001	11%	0.33
≤ 8 wks	11	1.09 [0.44, 1.75]	0.001	56%	
**Stem cell origin**					
Syngeneic	16	1.18 [0.67, 1.69]	< 0.001	48%	0.32
Allogeneic	2	1.92 [0.93, 2.92]	< 0.001	0%	
Xenogenic	1	1.93 [0.46, 3.41]	0.01	—	

BMD, bone mineral density; RCT, randomized controlled trials; BMCs, bone marrow cells; BMSCs, bone marrow mesenchymal stem cells; ADSCs, adipose-derived stem cells; HUCBDCs, human umbilical cord blood-derived CD34+ cells; ESCs, embryonic stem cells; IBM, intra-bone marrow; ITV, intra-tail venous; ICV, intra-cardio ventricular; CT, computed tomography; SMD, standard mean difference; CI, confidence interval.

### Sensitivity analyses and publication bias

**Fig 3:**
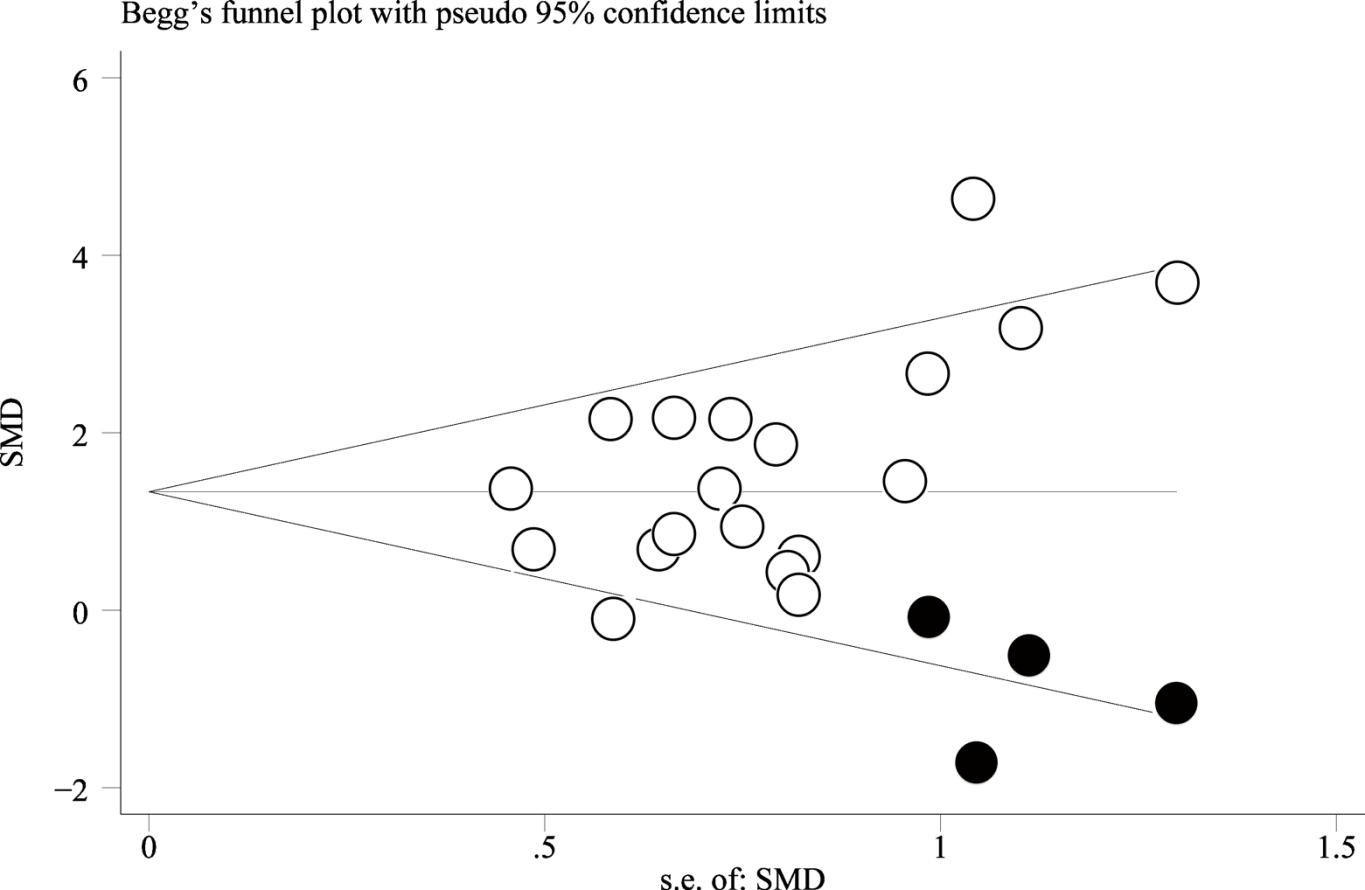
Funnel plot using ‘trim and fill’ method for meta-analysis of standardized mean difference (SMD) of bone mineral density for animals assigned to the stem cell based treatment and the control groups. The unfilled data points represent the identified studies included in the meta-analysis, and the black dots represent the imputed missing studies after adjustment for publication bias. *s*.*e*. *of SMD; standard error of the standardized mean difference*

Sensitivity analysis was performed by sequentially excluding one comparison at a time from the analysis, which revealed no significant effect on the results of the meta-analysis (data not shown). The funnel plots (**Fig 3**) for the meta-analysis were asymmetrical on visual inspection, suggesting that possible publication bias may have existed among the included studies. Consistent with this finding, the results of Egger’s significance tests also indicated the existence of potential publication biases (Egger’s regression test *p* = 0.055). Subsequently, we performed “trim-and-fill” analysis which conservatively imputes four hypothetical negative unpublished studies in order to produce a symmetrical funnel plot. The pooled analysis incorporating the hypothetical studies continued to show a statistically improved BMD in osteoporotic animals assigned to the stem cell therapy group, as compared to the controls (23 comparisons, SMD = 1.24, 95% CI: 0.32–2.16, *p* < 0.001).

## Discussion

In this meta-analysis, by pooling the results of available controlled preclinical studies, we found that stem cell transplantation was associated with significantly improved BMD as compared with controls in animal models of osteoporosis. The beneficial effects of stem cell based therapy on BMD seemed not to be significantly influenced by the models used, delivery route or amounts of stem cells used. Moreover, results of our meta-analysis indicate that stem cells from various sources, including BMCs, BMSCs, ADSCs, and HUCBDCs had a comparable effect on BMD, with the only exception of ESCs which did not appear to improve BMD. Overall, these results suggest a potential role of stem cell based therapy as a therapeutic agent in patients with osteoporosis.

Although the concept of stem cell based therapy for osteoporosis has evoked considerable interest over the years, to the best of our knowledge, no clinical trial in humans has been published. During the past decade, the therapeutic role of stem cell based strategy in osteoporosis has been studied in animal models. However, a quantitative evaluation of the results of these studies has been exceedingly rare. Since the preclinical studies often serve as a basis for future clinical trials, we performed a meta-analysis of the controlled studies to evaluate the effect of stem cell based therapy on BMD in animal models of osteoporosis, in order to gain insights from the currently available evidence [31]. Current evidence from animal studies appear to uphold the promise of stem cell based therapy for treatment of osteoporosis.

We chose BMD as the outcome of interest as it is known to correlate well with the severity of osteoporosis [25], and has also been shown to be a reliable predictor for the risk of fracture in patients with osteoporosis [32, 33]. Therefore, the observed improvement of BMD after stem cell transplantation strongly suggests that the therapeutic effect of stem cells on BMD may translate into reduced incidence of fractures, which is of immense clinical import.

The mechanisms underlying the potential benefits of stem cell based therapy on BMD mainly depend on their regenerative characteristics and their efficacy for restoration of bone formation ability [34]. Typically, after transplantation via a systemic or a topical route, stem cells migrate to the injured region and exert their regenerative function by modifying the environment and by recruiting resident cells. Besides the traditional mechanisms, recent studies have indicated that epigenetic regulation may also be involved in the stem cell mediated bone regeneration processes, suggesting that the mechanisms underlying the benefits of stem cell on bone formation are complex [35]. Further studies are needed to ascertain the fate and precise mechanisms underlying the benefits of stem cell transplantation in animal models of osteoporosis.

On stratified analyses, the benefit of stem cell based therapy on BMD seemed to be not affected by the characteristics like study design, animals and models used, dosages and delivery route as well as the BMD measurement methods used, which appears to reflect the robustness of the results. Moreover, sensitivity analyses by omitting one study a time did not significantly change the results, showing that the overall conclusion of the meta-analysis was not primarily contributed by any single study. However, the results of stratified analyses did not support a beneficial role of ESCs transplantation on BMD. This particular aspect should be evaluated in further studies, since ESCs were used only in one study.

Some key limitations of our meta-analysis should be considered when interpreting the results. Firstly, although we found that stem cell based therapy could improve BMD in animals of osteoporosis, we cannot infer that this leads to a beneficial effect on bone strength, since the influence of stem cell based therapy on the microarchitecture of the newly formed bones was not studied as it was beyond the scope of the current meta-analysis. Future studies are warranted to explore this aspect in greater detail. In addition, the overall quality of the included studies was modest (with a mean Jadad Sore: 2), and none of the studies were blinded. Moreover, the studies which were included in our meta-analysis generally used small animal models of osteoporosis. Therefore, randomized and blinded controlled studies in large animal models of osteoporosis are warranted to confirm our conclusions [36]. Secondly, considerable heterogeneity was detected among the included animal studies, and our pilot stratified analyses did not account for the heterogeneity. Although several factors such as variability in research methods, characteristics of laboratory animals, interventions, and outcome measures are liable to contribute to the heterogeneity in meta-analysis of animal studies, a more robust study design that allows for controlling the source of heterogeneity may improve the validity of the results. Besides, the origin of the grafts may also contribute to the heterogeneity among the included studies, although our exploratory subgroup analysis did not support a significant influence of the origin of the stem cells on their effect on BMD. Moreover, other factors including the ages of donors and recipients may also have a significant influence on the BMD. We were not able to evaluate the influence of ages of donors and recipients on BMD, because animals from different species were included and comparisons between ages of animals from different species are subjective. In addition, the possible role of immune responses on the effects of stem cell based therapy on BMD, particularly in those with allogeneic transplantations, may be more significant. However, we were unable to evaluate the potential influence of immune responses or the application of strategies against the rejection reaction since these data were rarely reported in the studies included in this meta-analysis. The influences of ages of donors and recipients, potential immune response, as well as the application of strategies against the rejection reaction deserve further investigations. Thirdly, we used BMD as the outcome of interest. Although its clinical relevance has been validated, the effect of stem cell based therapy on clinical outcomes such as the incidence of fractures probably deserves further observation. None of the included studies used autologous stem cell transplantation. Future studies are needed to determine the role of autologous stem cell transplantation for osteoporosis, which may be of more clinical relevance. Lastly, there were indications of publication bias affecting the results of this meta-analysis. This is a common phenomenon that generally affects the meta-analysis of animal studies, since the animal studies with positive results are more likely to be published [37, 38]. We addressed this issue by hypothetically imputing unpublished negative studies using “trim and fill” method, and found that the pooled results were not significantly affected by the publication bias.

In conclusion, results of our meta-analysis showed that stem cell transplantation was associated with significantly improved BMD as compared to that observed in controls in animal models of osteoporosis. These results suggest that stem cell based strategies may become a potential therapy for osteoporosis and further clinical studies are warranted.

## Supporting Information

S1 PRISMA Checklist (DOC)

S1 PRISMA Flow Diagram (DOC)

## Abbreviations

ADSCs: adipose-derived stem cells
BMCs: bone marrow cells
BMD: bone mineral density
BMSCs: bone marrow mesenchymal stem cells
CI: confidence interval
CT: computed tomography
ESCs: embryonic stem cells
HUCBDCs: human umbilical cord blood-derived CD34+ cells
IBM: intra-bone marrow
ICV: intra-cardio ventricular
ITV: intra-tail venous
qCT: quantitative CT
RCT: randomized controlled trial
SD: standard deviation
SMD: standard mean difference
