# Supplementary material for: Effect of Stem Cell Therapy on Bone Mineral Density: A Meta-Analysis of Preclinical Studies in Animal Models of Osteoporosis
Source: PLoS One. 2016 Feb 16;11(2):e0149400. doi: 10.1371/journal.pone.0149400 (PMC4755606; doi:10.1371/journal.pone.0149400)
Supplement: S1 PRISMA Flow Diagram — (DOC) [file pone.0149400.s002.doc]

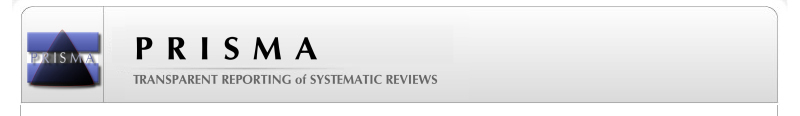
**PRISMA 2009 Flow Diagram**

**Screening**

**Included**

**Eligibility**

**Identification**

Records identified through database searching
(n = 232)

Additional records identified through other sources
(n = 0)

Records after duplicates removed
(n = 216)

Records screened
(n = 216)

Records excluded
(n = 194)

Full-text articles assessed for eligibility
(n = 22)

Full-text articles excluded, with reasons
(n = 10)

Studies included in qualitative synthesis
(n = 12)

Studies included in quantitative synthesis (meta-analysis)
(n = 12)
